# Minimally Invasive Stabilization Versus Open Surgery for Spinal Metastases: A Retrospective Study Utilizing Propensity Score Matching and Weighting Sensitivity Analyses

**DOI:** 10.3390/jcm15041653

**Published:** 2026-02-22

**Authors:** Kamil Krystkiewicz, Aleksander Kowal, Agata Krajniak, Łukasz Kuncman, Marcin Tosik

**Affiliations:** 1 Department of Neurosurgery and Neurooncology, Copernicus Memorial Hospital, 93-513 Lodz, Poland; aleksanderwkowal@gmail.com (A.K.); agata.krajniak@gmail.com (A.K.); marcin.tosik@wp.pl (M.T.); 2 Department of Radiotherapy, Medical University of Lodz, 93-513 Lodz, Poland; lukaszkuncman@gmail.com

**Keywords:** surgical site infection, metastatic spine disease, minimally invasive spine surgery, surgery complications

## Abstract

**Background**: Minimally invasive spinal stabilization (MISS) is increasingly used in metastatic spine surgery, but comparative evidence vs. open posterior stabilization (OPEN) remains limited. We compared perioperative outcomes, focusing on wound-related morbidity. **Methods**: This retrospective single-center cohort included 71 patients undergoing posterior stabilization for spinal metastases (MISS n = 45; OPEN n = 26). Wound-healing disorder was the primary endpoint. Groups were compared using nonparametric exact tests; adjusted and propensity score analyses were performed to assess robustness. **Results**: Baseline SINS, operated segment, and instrumented levels were comparable. BMI was higher in MISS (25.8 [24.0–29.7] vs. 22.1 [20.0–24.9] kg/m^2^; *p* = 0.001), and urgent admissions were more frequent in OPEN (42.3% vs. 11.1%; *p* = 0.006). Wound-healing disorders occurred in 6.7% (3/45) of the MISS group vs. 30.8% (8/26) of the OPEN group. (*p* = 0.014; crude RR 4.62, 95% CI 1.34–15.88). After adjustment for admission type, BMI, and ECOG (n = 65), the association was attenuated (adjusted RR 1.80, 95% CI 0.24–13.68; *p* = 0.572). SSI occurred in 1/45 (2.2%) MISS vs. 5/26 (19.2%) OPEN (*p* = 0.022). Estimated blood loss was similar between groups (MISS: 500 [350–800] vs. OPEN: 600 [500–700] mL; *p* = 0.357). The median length of stay was shorter in the MISS group, though this did not reach statistical significance. In trimmed IPTW (64 complete cases), OPEN remained associated with higher weighted risk (RR 1.91, 95% CI 0.42–8.65; *p* = 0.403). **Conclusions**: OPEN surgery was associated with higher unadjusted wound-related morbidity than MISS, while blood loss did not differ between approaches. Length of stay tended to be shorter after MISS, but analyses were underpowered.

## 1. Introduction

Minimally invasive spine surgery is widely adopted in modern spine care. In degenerative spine disease, minimally invasive techniques are well established and associated with reduced paraspinal soft-tissue disruption, lower wound-related morbidity, including fewer surgical site infections, and shorter hospital stays compared with conventional open approaches [1,2,3,4,5]. Spine oncology is a distinct clinical setting with different goals, higher baseline patient frailty, and frequent multimodal treatment pathways [6]. Patients with metastatic spine disease often receive systemic therapies that can impair immune function and wound healing, and postoperative radiotherapy is commonly required for local control; consequently, postoperative wound complications may delay adjuvant treatment and compromise timely continuation of oncologic care [7,8,9]. Overall, complication rates after oncologic spine procedures are higher than those reported in degenerative disease or trauma, making strategies that minimize surgical trauma particularly relevant in this population [10,11]. These considerations provide a rationale for extending minimally invasive techniques to metastatic spine surgery to reduce wound morbidity and facilitate uninterrupted oncologic treatment [12]. Although emerging studies suggest potential perioperative benefits of minimally invasive approaches in patients with spinal metastases, comparative evidence remains limited and heterogeneous, and the optimal role of minimally invasive stabilization in this setting remains unclear [11,13]. Therefore, this study aimed to compare minimally invasive spinal stabilization (MISS) with open posterior stabilization (OPEN) for metastatic spine disease using propensity score methods (matching and weighting) and prespecified sensitivity analyses to improve comparability between groups and assess the robustness of the findings.

## 2. Materials and Methods

**Figure 1:**
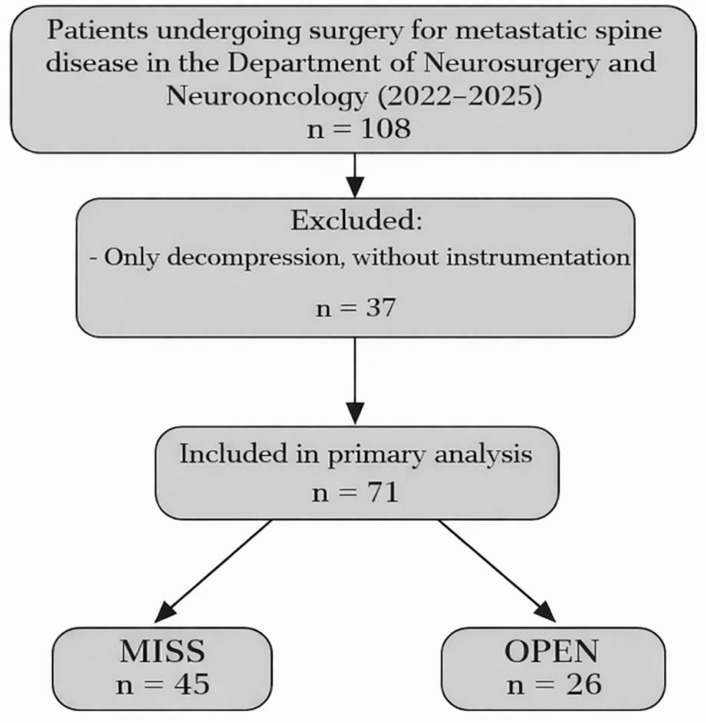
Patient selection flowchart. Flow diagram showing patient inclusion for the retrospective single-center cohort.

**Figure 2:**
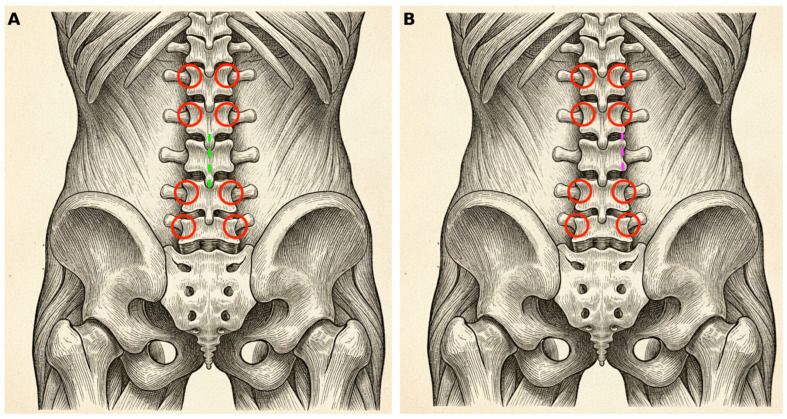
Schematic illustration of incision planning for percutaneous fixation and a separation procedure. Red circles mark the planned trajectories of percutaneous pedicle screws, along with their working sleeves. The dashed line indicates the skin incision required for the separation. (**A**) The midline approach, used when bilateral separation and decompression are required. (**B**) The unilateral approach, used when the tumor is unilateral and confined to the pedicle, facet joints, and adjacent portions of the vertebral body.

This retrospective, single-center cohort study included consecutive patients who underwent surgery for spinal metastases at the Department of Neurosurgery and Neurooncology, Copernicus Memorial Hospital, Lodz, Poland. Patients underwent posterior stabilization using either minimally invasive spinal stabilization (MISS) or open posterior stabilization (OPEN). The final cohort comprised 71 patients (MISS n = 45; OPEN n = 26) (Figure 1). In the MISS group, pedicle screws were inserted percutaneously with minimal soft-tissue retraction, using fluoroscopic guidance and muscle-sparing trajectories. When separation surgery was required, decompression was achieved through small skin incisions with limited subperiosteal dissection. For bilateral separation, decompression was performed through a small, separate midline incision centered over the affected level, allowing laminectomy, bilateral facetectomy, and circumferential epidural decompression with bilateral resection of the posterior longitudinal ligament to achieve separation of the neural elements from the tumor. For unilateral epidural involvement, a unilateral minimally invasive approach was used, with tubular access created between the ipsilateral screws; decompression and separation were performed through this corridor after limited soft-tissue dilation (Figure 2). In contrast, OPEN procedures were performed through a conventional midline approach with open posterior exposure, muscle detachment, and standard posterior stabilization, with decompression performed through the same open exposure when indicated. Baseline variables included age, body mass index (BMI), smoking history, admission type (elective vs. urgent), operated spinal segment, number of instrumented levels, Eastern Cooperative Oncology Group (ECOG) performance status (analyzed as 0–II vs. III–IV), Frankel grade, total Spinal Instability Neoplastic Score (SINS) and SINS components, and epidural spinal cord compression (ESCC) grade. Operative variables included operative time, implant material (titanium vs. carbon), procedure type, use of a vertebral body prosthesis, and instrumentation failure.

The primary endpoint was wound-healing disorder, defined as any postoperative wound complication, including surgical site infection (SSI); SSI was additionally analyzed as a subset outcome. Continuous variables are reported as median (interquartile range) and compared using the Mann–Whitney U test, while categorical variables are reported as counts (percentages) and compared using Fisher’s exact test or chi-square test as appropriate (two-sided *p* < 0.05). Crude associations for the primary endpoint were summarized using risk ratios (RR) with 95% confidence intervals. Adjusted analyses used modified Poisson regression with robust standard errors to estimate adjusted RR, including admission type, BMI, and ECOG (III–IV vs. 0–II). Because of sparse events, Firth’s penalized logistic regression with the same covariates was performed as a sensitivity analysis and reported as odds ratios (OR). Prespecified sensitivity analyses included elective admissions only and a separation surgery-only subset excluding corpectomy. Propensity score matching (PSM) was performed using 1:1 nearest-neighbor matching without replacement based on age, BMI, ECOG, total SINS, instrumented levels, admission type, and ESCC; paired Wilcoxon signed-rank and exact McNemar tests were used for matched pairs. Inverse probability of treatment weighting (IPTW) used stabilized weights derived from logistic regression including age, BMI, ECOG, instrumented levels, admission type, ESCC (low 0–1 vs. high 2–3), and SINS (≤6, 7–12, >12); weights were trimmed at the 95th percentile, balance was assessed using standardized mean differences (SMD) and Love plots, and the IPTW effect was estimated using robust Poisson regression. To account for the hypervascular nature of renal cell carcinoma (RCC) metastases, additional models adjusted for RCC histology were fitted: estimated blood loss (EBL) was analyzed using a Gamma GLM with log link (reporting ratios), and red blood cell (RBC) transfusion (yes/no) using robust modified Poisson regression (reporting RR). All analyses were performed in Python 3.11.2 (core packages included pandas, numpy, scipy, statsmodels, matplotlib, openpyxl, and python-docx).

This retrospective, non-interventional study was conducted in accordance with the Declaration of Helsinki and was based solely on routinely collected clinical data. Under the current Polish regulatory framework, retrospective analyses of existing medical records that do not involve any additional procedures do not require formal approval by a bioethics (IRB) committee. During hospitalization, patients provide standard written consent that allows the use of their medical data for scientific purposes; all data were analyzed in a de-identified form.

## 3. Results

### 3.1. Patient Characteristics

A total of 71 patients were included (MISS, n = 45; OPEN, n = 26). Baseline clinical characteristics are summarized in Table 1. Age did not differ between groups (MISS: 68.0 [IQR 60.0–72.0] vs. OPEN: 65.0 [57.0–72.8] years; *p* = 0.685). ECOG performance status, dichotomized into 0–II vs. III–IV, showed no statistically significant difference (MISS: 82.2% vs. OPEN: 65.4% in 0–II; *p* = 0.150). The overall SINS was comparable (MISS: 10.0 [9.0–12.0] vs. OPEN: 10.0 [8.0–11.0]; *p* = 0.476), as were instrumented levels (MISS: 5.0 [4.0–5.0] vs. OPEN: 5.0 [3.2–5.0]; *p* = 0.457) and the distribution of ESCC grades (*p* = 0.517). Operated spinal segment distribution did not significantly differ (*p* = 0.261). BMI was higher in the MISS group (25.8 [24.0–29.7] vs. 22.1 [20.0–24.9] kg/m^2^; *p* = 0.001). Admission type differed significantly, with urgent admissions more common in the OPEN group (42.3% vs. 11.1%; *p* = 0.006). Frankel grade distribution was similar (*p* = 0.816), and smoking history did not differ between groups (*p* = 0.705). The distribution of primary tumor histology is summarized in Table 1. Renal cell carcinoma (RCC) was the most frequent histology and was similarly represented in both groups (MISS: 10/45, 22.2% vs. OPEN: 8/26, 30.8%). Breast, lung, prostate, colorectal, thyroid, melanoma, lymphoma, and other less frequent primaries were grouped accordingly, while rare or heterogeneous diagnoses were aggregated under “Others” to improve interpretability. No statistically significant between-group differences were observed in the distribution of histology categories (all exploratory Fisher’s exact tests *p* > 0.05).

### 3.2. SINS Components

The distribution of individual SINS components is presented in Table 2. Lesion location, pain, bone lesion quality, spinal alignment, vertebral body collapse, and posterior element involvement did not differ significantly between MISS and OPEN (all *p* > 0.05). In contrast, involvement of the posterolateral element showed a significantly different distribution between groups (*p* = 0.003).

### 3.3. Operative Treatment Characteristics and Length of Stay

Operative treatment characteristics are summarized in Table 3. Briefly, operative time, implant material, procedure type distribution (including separation surgery and corpectomy), use of vertebral body prosthesis, and instrumentation failure did not differ significantly between groups (all *p* > 0.05). Length of hospital stay in the overall cohort was numerically longer in the OPEN group but did not differ significantly (MISS: 7.5 [6.0–11.8] days vs. OPEN: 9.5 [5.0–14.5] days; *p* = 0.375).

### 3.4. Wound-Healing Disorder

Wound-healing disorder occurred in 3/45 (6.7%) patients in the MISS group and 8/26 (30.8%) in the OPEN group (Fisher’s exact *p* = 0.014), corresponding to a crude RR of 4.62 (95% CI 1.34–15.88) for OPEN vs. MISS (Table 4). In a robust Poisson regression model adjusted for admission type, BMI, and ECOG, the association was attenuated and no longer statistically significant (adjusted RR 1.80, 95% CI 0.24–13.68; *p* = 0.572; n = 65). Firth’s penalized logistic regression yielded consistent results (adjusted OR 2.07, 95% CI 0.30–14.18; *p* = 0.458; n = 65). Surgical site infection, analyzed as a subset of wound-healing disorder, occurred in 1/45 (2.2%) MISS cases and 5/26 (19.2%) OPEN cases (Fisher’s exact *p* = 0.022).

### 3.5. Sensitivity Analyses

#### 3.5.1. Elective Admissions Only

Because admission type differed substantially between groups and urgent surgery may independently increase the risk of postoperative wound complications, a predefined sensitivity analysis was performed, restricted to elective admissions. In this elective-only cohort, wound-healing disorder occurred in 0/40 (0.0%) MISS patients and 4/15 (26.7%) OPEN patients (Fisher’s exact *p* = 0.004), with a crude RR of 23.06 (95% CI 1.32–404.34). Because there were no events in the MISS elective subgroup, adjusted modified Poisson regression produced unstable and non-interpretable RR estimates; therefore, adjustment was performed using Firth’s penalized logistic regression including BMI and ECOG (III–IV vs. 0–II), showing a similar direction of effect without reaching statistical significance (adjusted OR 13.61, 95% CI 0.79–235.56; *p* = 0.073; n = 50).

#### 3.5.2. Separation Surgery Only (Excluding Corpectomy)

Because corpectomy procedures are more complex and typically longer operations, an additional analysis excluded all corpectomy cases and was restricted to separation surgeries only (MISS, n = 24; OPEN, n = 9). Operative time did not differ significantly between groups (*p* = 0.517). Wound-healing disorder occurred in 1/24 (4.2%) MISS cases and 0/9 (0.0%) OPEN cases (*p* = 1.000). These results should be interpreted cautiously due to very low event counts and a limited sample size.

### 3.6. Propensity Score Analyses

**Figure 3:**
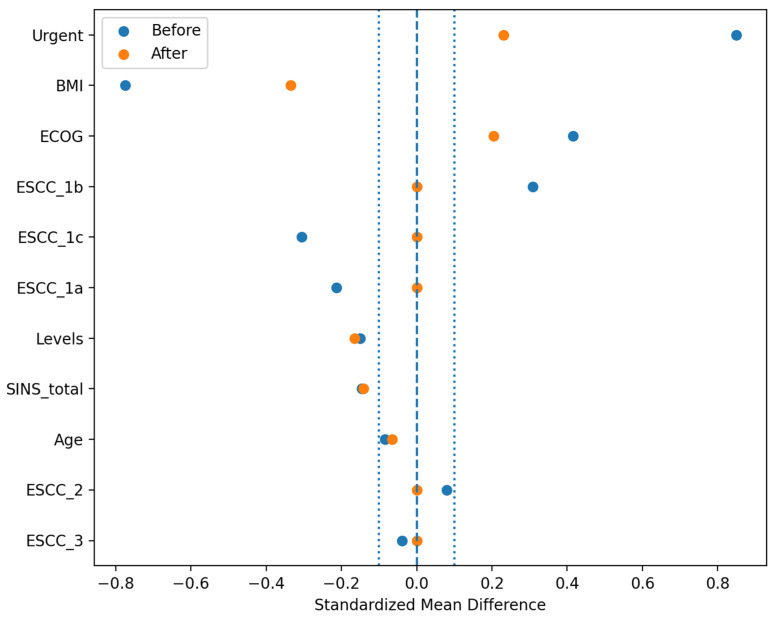
Covariate balance before and after propensity score matching (PSM). Love plot presenting standardized mean differences (SMD) for baseline covariates between the OPEN and MISS groups before matching and after 1:1 nearest-neighbor matching without replacement. Baseline covariates included body mass index (BMI), Eastern Cooperative Oncology Group performance status (ECOG), epidural spinal cord compression grade (ESCC; Bilsky scale categories 1a–1c, 2, and 3), number of involved spinal levels (Levels), Spinal Instability Neoplastic Score (SINS total), and urgent surgery status (Urgent). Vertical reference lines mark balance thresholds: the dashed line indicates perfect balance (SMD = 0), whereas the dotted lines indicate the commonly used criterion for acceptable balance (|SMD| = 0.1).

In the propensity score matched cohort (1:1 nearest neighbor matching without replacement; covariates: age, body mass index, ECOG performance status, total SINS, number of instrumented levels, admission type [urgent vs. elective], and ESCC category), 18 matched pairs were obtained (OPEN n = 18; MISS n = 18) (Figure 3). Operative time was numerically longer in the OPEN group but did not differ significantly (MISS: 190.0 [142.5–235.0] min vs. OPEN: 205.0 [180.0–235.0] min; paired Wilcoxon *p* = 0.297). Length of hospital stay was numerically higher in the OPEN group without reaching statistical significance (MISS: 7.0 [6.0–11.2] days vs. OPEN: 10.5 [6.0–15.8] days; paired Wilcoxon *p* = 0.347). Wound-healing disorder occurred in 1/18 (5.6%) MISS cases and 4/18 (22.2%) OPEN cases; although not statistically significant (exact McNemar *p* = 0.375), the direction of effect favored MISS.

**Figure 4:**
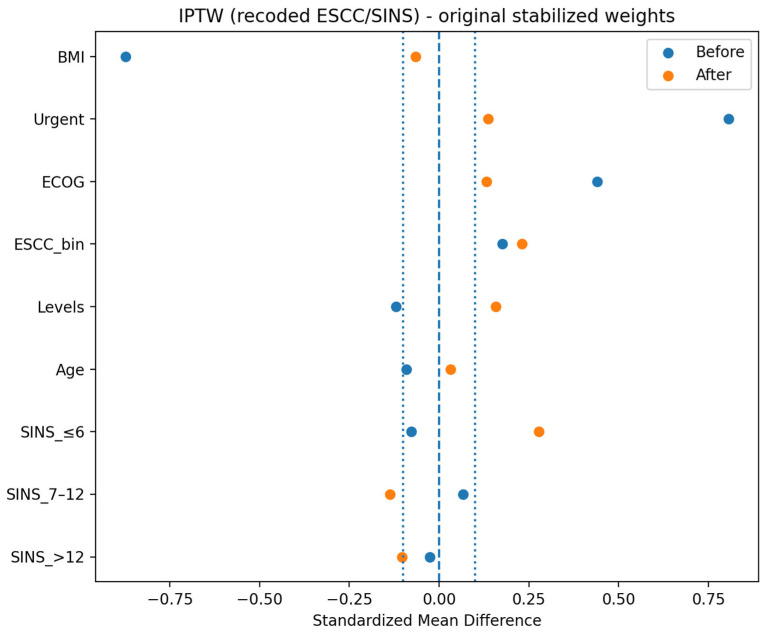
Covariate balance before and after IPTW with weight trimming (95th percentile cap). Love plot showing SMD before weighting and after applying IPTW, with trimmed weights capped at the 95th percentile to reduce the influence of extreme weights. Covariates and coding are the same as in Figure 3. Vertical reference lines indicate SMD = 0.1.

In an inverse probability of treatment weighting (IPTW) sensitivity analysis, propensity scores were estimated using logistic regression including age, body mass index, ECOG performance status, number of instrumented levels, admission type (urgent vs. elective), ESCC recoded as low grade (0–1) vs. high grade (2–3), and total SINS categorized as ≤6, 7–12, and >12. Stabilized IPTW was applied in 64 complete cases (OPEN n = 20, MISS n = 44). We capped weights at the 95th percentile (cap = 2.499), thereby improving covariate balance (the maximum SMD after IPTW decreased from 0.279 to 0.216). In the trimmed IPTW model, weighted wound-healing disorder risk was 15.1% in the OPEN group and 8.0% in the MISS group, corresponding to an IPTW-adjusted RR of 1.91 (95% CI 0.42–8.65; *p* = 0.403) (Figure 4). In the same trimmed IPTW analysis, the weighted length of hospital stay was numerically longer in the OPEN group (weighted median 11.2 [6.0–15.7] days) than in the MISS group (7.4 [6.0–12.0] days), but this difference was not statistically significant (weighted model *p* = 0.197). Overall, although the adjusted and propensity score analyses were not statistically significant, the direction of effects consistently favored MISS, suggesting limited power due to the small number of events.

### 3.7. Blood Loss and Transfusions

**Figure 5:**
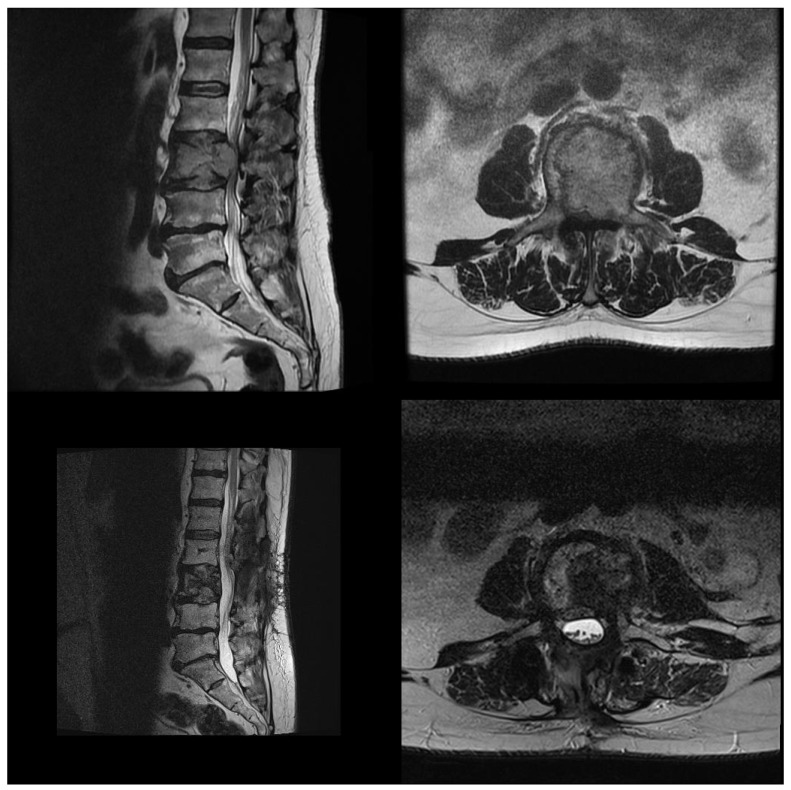
Illustrative case of metastatic renal cell carcinoma with an L3 vertebral body metastasis causing high-grade epidural spinal cord compression (ESCC grade 3). The patient was treated with minimally invasive transpedicular stabilization using a carbon fiber–reinforced PEEK system, and a separation procedure was performed through a small midline incision. The lower panels show postoperative follow-up MRI and MRI after stereotactic body radiotherapy (SBRT) (6 weeks after surgery and 4 weeks after SBRT). Imaging demonstrates satisfactory decompression with no residual epidural tumor within the spinal canal and a clearly visible cauda equina within the dural sac.

In crude comparisons, estimated blood loss (EBL) was similar between groups (MISS: 500 [350–800] mL, n = 36 vs. OPEN: 600 [500–700] mL, n = 18; *p* = 0.357). Red blood cell (RBC) transfusion rates were also comparable (MISS: 13/40 [32.5%] vs. OPEN: 10/25 [40.0%]; *p* = 0.600), and the distribution of transfused units did not differ (*p* = 0.727). Because renal cell carcinoma (RCC) metastases are associated with hypervascularity and may increase intraoperative bleeding risk despite the embolization procedures, we performed additional analyses controlling for RCC histology. RCC prevalence did not differ between surgical approaches (MISS: 10/42 [23.8%] vs. OPEN: 8/26 [30.8%]; Fisher’s exact *p* = 0.579). In RCC-adjusted models, OPEN was not associated with higher bleeding or transfusion risk. In a Gamma generalized linear model (log link) for EBL, the OPEN vs. MISS effect was non-significant (ratio 0.98, 95% CI 0.69–1.40; *p* = 0.928), whereas RCC histology was independently associated with higher blood loss (ratio 1.64, 95% CI 1.13–2.37; *p* = 0.009). Similarly, in a robust modified Poisson model for any RBC transfusion, OPEN did not increase transfusion risk after adjusting for RCC (RR 1.13, 95% CI 0.58–2.20; *p* = 0.712); results were consistent in a logistic regression sensitivity analysis (OR 1.23, 95% CI 0.42–3.55; *p* = 0.708). An illustrative case demonstrating minimally invasive transpedicular carbon fiber–reinforced PEEK fixation combined with a limited midline separation procedure for an L3 metastasis (ESCC grade 3) is presented in Figure 5.

### 3.8. Reoperations and Readmissions

In our cohort, any reoperation occurred in 5/45 (11.1%) MISS vs. 4/26 (15.4%) OPEN patients (*p* = 0.716). Wound washout/debridement was required in 1/45 (2.2%) MISS and 4/26 (15.4%) OPEN cases (*p* = 0.057). Removal of instrumentation due to wound complications was rare (1/45 [2.2%] MISS vs. 0/26 [0.0%] OPEN; *p* = 1.000). Instrumentation failure and revision for failure were similarly infrequent (failure: 2/45 [4.4%] vs. 1/26 [3.8%]; revision: 2/45 [4.4%] vs. 1/26 [3.8%]; both *p* = 1.000). Reoperation for local tumor recurrence occurred in 1/45 (2.2%) MISS vs. 1/26 (3.8%) OPEN patients (*p* = 1.000). Regarding postoperative readmissions, there were 2 readmissions in the MISS group (2/45, 4.4%) and 2 in the OPEN group (2/26, 7.7%). In the MISS cohort, readmissions were due to hardware removal for a wound-related problem (n = 1) and reoperation for local tumor recurrence (n = 1). In the OPEN cohort, readmissions were due to wound debridement (n = 1) and reoperation for local tumor recurrence (n = 1). The overall readmission rate did not differ significantly between groups (Fisher’s exact *p* = 0.616).

## 4. Discussion

Surgery for metastatic spine disease is frequently burdened by postoperative complications, reflecting the frailty of this patient population and the need to integrate surgical care with systemic therapy and radiotherapy. Because wound-related morbidity can delay recovery and interrupt planned oncologic treatment, strategies that reduce perioperative complications are particularly relevant in spine oncology. MISS is an established concept in degenerative indications, but its role in metastatic spine surgery is less uniform and often depends on case complexity and urgency [11]. In this context, our findings suggest a generally favorable perioperative profile for MISS compared with open posterior stabilization, with a consistent direction of effect toward fewer wound-related problems across comparative analyses, although statistical significance was not consistently achieved.

Several meta-analyses and comparative cohorts in metastatic spine surgery have suggested that minimally invasive or percutaneous posterior stabilization is associated with lower estimated blood loss (EBL) and, in some reports, fewer transfusions than open posterior instrumentation [13]. These differences are most often attributed to reduced muscle dissection and a smaller exposure during the instrumentation phase. However, our data add an important nuance: after explicitly accounting for renal cell carcinoma (RCC), we still observed no meaningful differences between MISS and OPEN in either EBL or red blood cell transfusion risk. A likely explanation is procedural: in metastatic decompression surgery, a substantial proportion of blood loss originates from the tumor-related decompression/resection component rather than from the posterior stabilization itself. When decompression and tumor handling are performed in both MISS and OPEN (with similar oncologic intent), any reduction in bleeding attributable solely to a less invasive stabilization corridor may be diluted. This interpretation is consistent with broader evidence showing that blood loss and transfusion requirements in metastatic spine tumor surgery are driven primarily by tumor biology (including hypervascular primaries), operative time, and the overall extent/type of surgery [14]. RCC is a prototypical hypervascular metastasis and a well-established determinant of higher bleeding risk, often prompting dedicated blood-sparing strategies such as preoperative embolization [15]. In our cohort, RCC was independently associated with higher blood loss, yet the approach (OPEN vs. MISS) was not, and transfusion rates remained comparable after adjustment. Taken together, these findings suggest that, in our setting, the dominant drivers of bleeding were the resection/decompression phase and tumor vascularity, which were present across approaches, rather than the stabilization technique per se.

In our cohort, length of hospital stay was numerically shorter after minimally invasive spinal stabilization (MISS) than after open posterior stabilization (OPEN) (median 7.0 vs. 10.5 days), although this difference did not reach statistical significance. Despite the lack of statistical significance, the magnitude of the observed difference suggests a clinically meaningful trend favoring MISS and is most consistent with limited power in a small, heterogeneous oncologic sample rather than clear equivalence between techniques. Importantly, hospital stay in metastatic spine surgery is strongly influenced by baseline acuity (e.g., urgent admissions), perioperative morbidity, and the need to resume systemic therapy and/or radiotherapy; therefore, residual confounding by indication may attenuate between-group comparisons even with adjustment. Prior comparative literature generally reports shorter hospitalization with minimally invasive or percutaneous posterior fixation strategies compared with open surgery, supporting the biological plausibility of faster recovery after reduced soft-tissue disruption and lower wound morbidity [13] and consistent with broader evidence syntheses in metastatic epidural spinal cord compression populations [10]. Similar findings have also been reported in comparative single-center cohorts of patients undergoing metastatic spine tumor surgery with percutaneous pedicle-screw fixation vs. open approaches [14].

In our study, surgical site infection (SSI) occurred in 1/45 (2.2%) MISS cases compared with 5/26 (19.2%) OPEN cases (*p* = 0.022), indicating a markedly lower infection burden with the minimally invasive approach. This finding is clinically relevant because SSI is one of the most consequential complications in spine surgery, frequently requiring prolonged antibiotics, wound care, and/or reoperation, which in turn extends hospital length of stay and increases overall treatment costs [16]. From an oncology perspective, preventing SSI is particularly valuable because infection-related delays in recovery can disrupt planned adjuvant radiotherapy or systemic therapy. The direction of our result is consistent with published comparative evidence in metastatic spine surgery, where percutaneous/minimally invasive posterior fixation strategies have been associated with lower infection rates and shorter hospitalization compared with open posterior instrumentation [13]. Mechanistically, this advantage is biologically plausible, as MISS reduces soft-tissue disruption and dead space around instrumentation, potentially lowering bacterial burden and improving wound healing conditions in a population with limited physiologic reserve. Finally, given that SSI is consistently linked to increased resource utilization and cost across spine surgery populations, the observed reduction in infections with MISS represents a meaningful potential value proposition beyond clinical outcomes alone [17]. Despite low event counts, secondary outcomes showed a consistent trend toward fewer wound-related reinterventions and readmissions after MISS than after OPEN, although these differences did not reach statistical significance and should be interpreted cautiously given limited statistical power.

The suitability of MISS techniques in metastatic spine disease, particularly in the presence of epidural involvement, should be discussed in the context of contemporary hybrid oncologic care rather than as a binary “MISS vs. OPEN” question. Current decision-making frameworks emphasize aligning the surgical corridor and extent of decompression with neurologic status, mechanical stability, tumor biology, and the planned adjuvant strategy, most commonly postoperative radiotherapy or stereotactic body radiotherapy (SBRT) [6]. Within this paradigm, MISS can be combined with targeted decompression via mini-open or tubular corridors to achieve the key oncologic objective of separation surgery: circumferential decompression of neural elements to enable safe, effective postoperative SBRT while avoiding unnecessary soft-tissue disruption [6,10,11,12]. This approach is particularly attractive in metastatic populations where wound morbidity may delay adjuvant radiation and compromise timely continuation of multimodal treatment [8,9]. At the same time, the literature also supports a balanced view: minimally invasive techniques are not intended for en bloc resections, and major deformity correction (e.g., pronounced kyphosis or sagittal imbalance) often necessitates open or hybrid strategies; thus, careful patient selection remains essential [11,12]. Importantly, fixation reliability in fragile oncologic bone is another key aspect of this discussion. Poor bone quality, frequently exacerbated by systemic therapies and radiotherapy, can challenge construct durability; however, modern strategies such as cement augmentation with fenestrated screws have shown encouraging mechanical reliability in compromised bone and provide a useful analog for understanding the low instrumentation failure rates observed in contemporary fixation constructs [18]. Overall, available evidence suggests that, for appropriately selected patients, minimally invasive corridors can deliver adequate decompression (including separation) and stabilization consistent with hybrid management goals, while potentially mitigating clinically consequential wound-related morbidity in spine oncology [10,11,12,13,14].

This retrospective single-center study includes a consecutive real-world cohort of patients undergoing surgery for metastatic spine disease and focuses on clinically meaningful hospitalization-related and perioperative outcomes. The analytical strategy was prespecified and strengthened by complementary approaches, including crude comparisons, multivariable adjustment, sensitivity analyses in more comparable subgroups, and propensity score methods (matching and weighting) with balance diagnostics. We also provided detailed baseline characterization (including SINS/ESCC), procedure mix, oncologic therapy exposure, and RCC-adjusted models for bleeding-related outcomes.

Several limitations should be acknowledged. The sample size was modest (n = 71), and wound events were infrequent, resulting in limited power and wide confidence intervals, particularly for adjusted and propensity analyses; therefore, the results should be considered exploratory. With 45 vs. 26 patients, the study was primarily able to detect only large between-group differences; smaller-to-moderate effects would be expected to remain statistically non-significant despite consistent estimates. Notably, the direction of effect was consistent across crude, adjusted, and propensity score analyses, supporting a reproducible trend that warrants confirmation in larger cohorts. Baseline imbalances (notably urgent admissions and BMI) raise the possibility of residual confounding despite adjustment and propensity methods. Procedure heterogeneity (stabilization-only and decompression-plus-stabilization, including separation surgery and corpectomy) may dilute approach-specific effects, even after subset analyses.

## 5. Conclusions

While crude analysis showed a lower rate of SSI in the MISS group, this association was attenuated after adjusting for baseline imbalances, suggesting that patient selection and urgency significantly contribute to postoperative morbidity. No differences were observed in estimated blood loss or transfusion-related outcomes between approaches, including after accounting for renal cell carcinoma histology. Although differences in other perioperative outcomes, including length of hospital stay, did not reach statistical significance, the direction and magnitude of effects consistently favored MISS, suggesting a potential benefit that may be underpowered in this sample. Overall, MISS appears to be a safe and valuable approach in appropriately selected metastatic spine cases, warranting confirmation in larger, prospective, multicenter studies.

## Figures and Tables

**Table 1:** Baseline clinical characteristics of the MISS and OPEN groups. Data are presented as median [interquartile range] or n (%). MISS, minimally invasive surgery; OPEN, open surgery; ECOG, Eastern Cooperative Oncology Group performance status; SINS, Spinal Instability Neoplastic Score; ESCC, Epidural Spinal Cord Compression; BMI, body mass index. *p*-values are from unadjusted between-group comparisons.

Variable	MISS (n = 45)	Open (n = 26)	*p*-Value
**Age** (**years**)	68.0 [60.0–72.0]	65.0 [57.0–72.8]	0.685
**ECOG**	0–II: 37 (82.2%) III–IV: 8 (17.8%)	0–II: 17 (65.4%) III–IV: 9 (34.6%)	0.15
**SINS total score**	10.0 [9.0–12.0]	10.0 [8.0–11.0]	0.476
**Operated spinal segment**	Thoracic: 29 (64.4%) Lumbar: 14 (31.1%) Cervical: 2 (4.4%)	Thoracic: 16 (61.5%) Lumbar: 6 (23.1%) Cervical: 4 (15.4%)	0.261
**Instrumented levels**	5.0 [4.0–5.0]	5.0 [3.2–5.0]	0.457
**ESCC**	1A: 1 (2.2%) 1C: 6 (13.3%) 2: 11 (24.4%) 3: 27 (60.0%)	1A: 1 (3.8%) 1B: 1 (3.8%) 1C: 2 (7.7%) 2: 7 (26.9%) 3: 15 (57.6%)	0.517
**BMI, kg/m^2^**	25.8 [24.0–29.7]	22.1 [20.0–24.9]	**0.001**
**Smoking history**	Never: 25 (55.6%) Current: 7 (15.6%) Former: 7 (15.6%) Unknown: 6 (13.3%)	Never: 12 (46.2%) Former: 7 (26.9%) Current: 4 (15.4%) Unknown: 3 (11.5%)	0.705
**Admission type**	Elective: 40 (88.9%) Urgent: 5 (11.1%)	Elective: 15 (57.7%) Urgent: 11 (42.3%)	0.006
**Frankel grade**	E: 29 (64.4%) D: 9 (20.0%) C: 4 (8.9%) B: 2 (4.4%) A: 1 (2.2%)	E: 13 (50.0%) D: 6 (23.1%) C: 4 (15.4%) B: 2 (7.7%) A: 1 (3.8%)	0.816
**Histopathology**			
**Renal cell carcinoma (RCC)**	10 (22.2%)	6 (23.1%)	0.733
**Breast cancer**	8 (17.8%)	8 (30.8%)	
**Lung cancer**	5 (11.1%)	3 (11.5%)	
**Adenocarcinoma (unknown, unspecified origin)**	3 (6.7%)	2 (7.7%)	
**Extraspinal sarcoma**	1 (2.2%)	2 (7.7%)	
**Colorectal cancer**	2 (4.4%)	3 (11.5%)	
**Melanoma**	1 (2.2%)	1 (3.8%)	
**Others**	15 (33.3%)	6 (23.1%)	
**Previous systemic treatment** **Chemotherapy** **Radiotherapy** **Immunotherapy**	13 (28.9%) 21 (46.7%) 1 (2.2%)	7 (26.9%) 6 (23.1%) 2 (7.7%)	0.144

**Table 2:** Distribution of SINS component categories in the MISS and OPEN groups. Values are presented as n (% within group). *p*-values refer to unadjusted between-group comparisons of the full category distribution (chi-square or Fisher’s exact test, as appropriate). SINS, Spinal Instability Neoplastic Score; MISS, minimally invasive surgery; OPEN, open surgery.

Variable	Category (Points)	MISS (n = 45)	OPEN (n = 26)	*p*-Value
**Location**	Rigid (S2–S5) (0)	0 (0.0%)	0 (0.0%)	0.942
	Semi-rigid (T3–T10) (1)	19 (42.2%)	11 (42.3%)	
	Mobile (C3–C6, L2–L4) (2)	9 (20.0%)	6 (23.1%)	
	Junctional (C0–C2, C7–T2, T11–L1, L5–S1) (3)	17 (37.8%)	9 (34.6%)	
**Pain**	Pain-free lesion (0)	4 (8.9%)	3 (11.5%)	0.928
	Occasional/non-mechanical pain (1)	0 (0.0%)	0 (0.0%)	
	Occasional/non-mechanical pain (2)	4 (8.9%)	2 (7.7%)	
	Mechanical pain (3)	37 (82.2%)	21 (80.8%)	
**Bone lesion**	Blastic (0)	3 (6.7%)	1 (3.8%)	0.122
	Mixed lytic/blastic (1)	6 (13.3%)	0 (0.0%)	
	Lytic (2)	36 (80.0%)	25 (96.2%)	
**Spinal alignment**	Normal alignment (0)	39 (86.7%)	19 (73.1%)	0.315
	De novo deformity (2)	5 (11.1%)	5 (19.2%)	
	Subluxation/translation (4)	1 (2.2%)	2 (7.7%)	
**Vertebral body collapse**	None (0)	4 (8.9%)	5 (19.2%)	0.318
	No collapse, >50% body involved (1)	17 (37.8%)	9 (34.6%)	
	<50% collapse (2)	14 (31.1%)	4 (15.4%)	
	>50% collapse (3)	10 (22.2%)	8 (30.8%)	
**Posterolateral involvement**	None (0)	5 (11.1%)	12 (46.2%)	**0.003**
	Unilateral involvement (1)	21 (46.7%)	9 (34.6%)	
	Bilateral involvement (3)	19 (42.2%)	5 (19.2%)	

**Table 3:** Operative treatment characteristics and postoperative complications in the MISS and OPEN groups. Values are presented as median [interquartile range] or n (% within group). *p*-values are from unadjusted between-group comparisons (Mann–Whitney U test for continuous variables; chi-square or Fisher’s exact test for categorical variables, as appropriate). MISS, minimally invasive surgery; OPEN, open surgery. Wound-healing disorder was defined as any postoperative wound complication and included surgical site infections.

Variable	Category	MISS (n = 45)	OPEN (n = 26)	*p*-Value
**Operative time (min)**		180.0 [145.0–230.0]	190.0 [166.2–218.8]	0.244
**Estimated blood loss (mL)**		500 [350–800]	600 [500–700]	
**Implant material**	Titanium	28 (62.2%)	12 (46.2%)	0.345
	Carbon	17 (37.8%)	14 (53.8%)	
	Unknown	1 (2.2%)	0 (0.0%)	
**Procedure type**	Separation surgery	24 (53.3%)	9 (34.6%)	0.063
	Corpectomy (piecemeal)	6 (13.3%)	7 (26.9%)	
	Corpectomy (en bloc)	0 (0.0%)	2 (7.7%)	
	Laminectomy	11 (24.4%)	8 (30.8%)	
	Stabilization only	4 (8.9%)	0 (0.0%)	
**Vertebral body prosthesis used**	Yes	4 (8.9%)	7 (26.9%)	0.085
	No	41 (91.1%)	19 (73.1%)	
**Wound-healing disorder**	Yes	3 (6.7%)	8 (30.8%)	**0.014**
	No	42 (93.3%)	18 (69.2%)	
**Surgical site infection**	Yes	1 (2.2%)	5 (19.2%)	**0.022**
	No	44 (97.8%)	21 (80.8%)	
**Instrumentation failure**	Yes	2 (4.4%)	1 (3.8%)	1.000
	No	43 (95.6%)	25 (96.2%)	
**Nosocomial infection**	Yes	7 (15.6%)	8 (30.8%)	0.225
	No	38 (84.4%)	18 (15.6%)	

**Table 4:** Comparison of effect estimates for the primary endpoint (wound-healing disorder) between MISS and OPEN. Effect estimates compare OPEN vs. MISS for wound-healing disorder, defined as any postoperative wound complication, including surgical site infection. Crude risk ratio (RR) was calculated from the 2 × 2 contingency table. Adjusted RR was estimated using modified Poisson regression with robust standard errors, adjusted for admission type (urgent vs. elective), body mass index, and ECOG performance status (III–IV vs. 0–II). Adjusted odds ratio (OR) was obtained from Firth’s penalized logistic regression with the same covariates. Values are presented as point estimates with 95% confidence intervals.

Model	Measure	Estimate	95% CI
**Crude RR (2 × 2)**	RR	4.62	1.34–15.88
**Adjusted RR (Poisson robust)**	RR	1.80	0.24–13.68
**Adjusted OR (Firth logistic)**	OR	2.07	0.30–14.18

## Data Availability

Data underlying the results presented in this article are not publicly available due to institutional regulations, but can be obtained from the corresponding author on reasonable request.

## Abbreviations

The following abbreviations are used in this manuscript:

BMI	body mass index
EBL	estimated blood loss
ECOG	Eastern Cooperative Oncology Group performance status
ESCC	epidural spinal cord compression
GLM	generalized linear model
IQR	interquartile range
IPTW	inverse probability of treatment weighting
LOS	length of stay
MISS	minimally invasive spinal stabilization
OPEN	open posterior stabilization
OR	odds ratio
PSM	propensity score matching
RBC	red blood cell
RCC	renal cell carcinoma
RR	risk ratio
SSI	surgical site infection
SINS	Spinal Instability Neoplastic Score
SMD	standardized mean difference
